# Macrophages inhibit extracellular hyphal growth of *A. fumigatus* through Rac2 GTPase signaling

**DOI:** 10.1128/iai.00380-23

**Published:** 2024-01-03

**Authors:** Christopher D. Tanner, Emily E. Rosowski

**Affiliations:** 1 Department of Biological Sciences, Clemson University, Clemson, South Carolina, USA; 2 Eukaryotic Pathogens Innovation Center, Clemson University, Clemson, South Carolina, USA; Tulane University, New Orleans, Louisiana, USA

**Keywords:** *Aspergillus fumigatus*, Rac GTPases, macrophages, zebrafish

## Abstract

Macrophages act as a first line of defense against pathogens. Against *Aspergillus fumigatus*, a fungus with pathogenic potential in immunocompromised patients, macrophages can phagocytose fungal spores and inhibit spore germination to prevent the development of tissue-invasive hyphae. However, the cellular pathways that macrophages use to accomplish these tasks and any roles macrophages have later in infection against invasive forms of fungi are still not fully known. Rac-family Rho GTPases are signaling hubs for multiple cellular functions in leukocytes, including cell migration, phagocytosis, reactive oxygen species (ROS) generation, and transcriptional activation. We therefore aimed to further characterize the function of macrophages against *A. fumigatus* in an *in vivo* vertebrate infection model by live imaging of the macrophage behavior in *A. fumigatus*-infected *rac2* mutant zebrafish larvae. While Rac2-deficient zebrafish larvae are susceptible to *A. fumigatus* infection, Rac2 deficiency does not impair macrophage migration to the infection site, interaction with and phagocytosis of spores, spore trafficking to acidified compartments, or spore killing. However, we reveal a role for Rac2 in macrophage-mediated inhibition of spore germination and control of invasive hyphae. Re-expression of Rac2 under a macrophage-specific promoter rescues the survival of *A. fumigatus*-infected *rac2* mutant larvae through increased control of germination and hyphal growth. Altogether, we describe a new role for macrophages against extracellular hyphal growth of *A. fumigatus* and report that the function of the Rac2 Rho GTPase in macrophages is required for this function.

## INTRODUCTION

In healthy people, innate immunity is generally sufficient to prevent invasive fungal infections, including those with *Aspergillus fumigatus*, *Candida albicans*, and *Cryptococcus* spp. (1, 2). Patients with impaired immune responses, including those with innate immune defects such as neutropenia, are at risk of invasive fungal disease (3). Existing anti-fungal drugs have low efficacy in patients, and ~50% of patients with invasive aspergillosis succumb to infection even with treatment (4). Host-directed therapies are a promising approach against many of these infections (5, 6), but a better understanding is required of the cellular mechanisms used by innate immune cells to control fungal pathogens.

*A. fumigatus* is a fungal species that causes >300,000 deaths per year worldwide (7). This fungus is present throughout the environment and infects humans as spores or conidia. In invasive aspergillosis, these spores germinate into hyphae and invade and destroy tissues causing devastating disease and death. In healthy individuals, neutrophils respond to the post-germination forms of the fungus and control and destroy invasive hyphae through well-characterized functions such as degranulation, reactive oxygen species (ROS) production, and NETosis (8–11). Macrophages, on the other hand, act as a first line of defense and can phagocytose and inhibit the germination of *A. fumigatus* spores (12–16). However, how well macrophages kill these spores *in vivo* and whether macrophages have any role in controlling extracellular post-germination fungal growth are still unclear. Additionally, while macrophages possess many anti-microbial functions, which pathways and mechanisms are most important against *A. fumigatus* is unknown.

One major hub of macrophage function and signaling are the Rac GTPases (17). Rac GTPases promote actin polymerization and can activate cell migration, phagocytosis, ROS production, and transcriptional activation (17–19). Rac2 is a leukocyte-specific Rac GTPase, and humans and mice with deficiencies in Rac2 are susceptible to infectious disease (20, 21). While Rac2 is absolutely required in neutrophils for basic cell functions including directed migration, the role of this GTPase in macrophages is less clear (21–23). We previously generated a *rac2* mutant zebrafish line and found that 30%–50% fewer macrophages respond to a wound or a *Pseudomonas aeruginosa* infection in *rac2* mutant larvae compared with control larvae (24). We also found that rescue of Rac2 expression specifically in macrophages is sufficient to significantly increase the survival of *rac2* mutant larvae infected with *P. aeruginosa* (24).

In this study, we utilized this larval zebrafish model to determine the function of Rac2 in macrophages against *A. fumigatus*, taking advantage of the live imaging capabilities of this model to visualize the requirement for Rac2 in macrophage responses in live intact hosts over a multi-day infection. The innate immune system of larval zebrafish is largely conserved with humans, and zebrafish have been used to model multiple human immunodeficiencies and infections (25–28) and to determine the function of neutrophils and macrophages (29). Here, we find that macrophages do not require Rac2 function to migrate to or phagocytose injected *A. fumigatus* spores. Consistent with previous results, ~50% of injected spores are killed by 2 days post injection (dpi) in wild-type animals (30, 31). However, we report that this killing is not always coincident with acidification of the intracellular spore compartment and that Rac2 is not required for either spore acidification or killing. In the absence of functional neutrophils, we report for the first time that macrophages can provide significant protection against invasive hyphal growth. While the mechanisms that macrophages use against extracellular fungal growth are still unclear, we find that Rac2 in macrophages is both necessary and sufficient to promote this hyphal control.

## RESULTS

### Rac2-deficient larvae are susceptible to *A. fumigatus* infection

**Fig 1:**
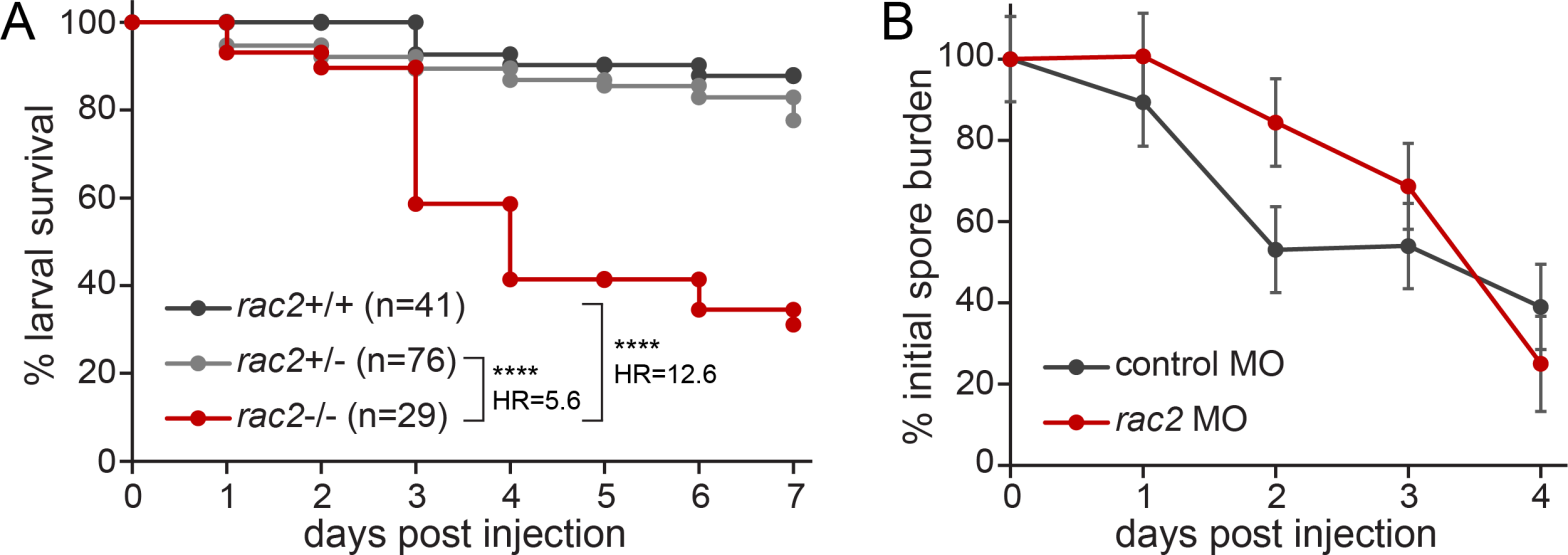
Rac2-deficient zebrafish larvae are susceptible to *A. fumigatus* infection. (A) *rac2*^+/−^ adult fish were crossed to each other, and the resulting larvae were injected with TBK1.1 (Af293) spores. Survival was monitored for 7 dpi, and larvae were genotyped at the conclusion of the experiment. Average injection CFUs = 46. Data represent three pooled replicates. *P* values and hazard ratios were calculated by Cox proportional hazard analysis. (B) Larvae previously treated with *rac2* or standard control MO were injected with TBK1.1 (Af293) spores. At 0–5 dpi, larvae were homogenized and plated to evaluate CFUs. CFU counts were normalized to the average injection dose for each group for each replicate. CFUs were evaluated from four to eight larvae per group per day per replicate. Average injection CFUs: control = 46, *rac2* = 56. Plotted data represent emmeans ± SEM from four pooled experiments; *P* values were calculated by analysis of variance (ANOVA).

We previously generated a *rac2^−/−^* zebrafish line and demonstrated susceptibility of *rac2^−/−^* larvae to both *Pseudomonas aeruginosa* and *Aspergillus fumigatus* infection (24). To re-confirm this susceptibility to *A. fumigatus* infection, we crossed *rac2*^+/−^ adult zebrafish to each other and infected 2-day post fertilization (dpf) larval progeny via hindbrain injection with spores of an Af293-derived strain of *A. fumigatus* through established methods (32). We monitored larval survival for 7 days and genotyped larvae at the conclusion of the experiment (Fig. S1A). As expected, *rac2^−/−^* larvae had significantly higher susceptibility to *A. fumigatus* infection compared with *rac2^+/−^* and *rac2^+/+^* siblings (Fig. 1A). To determine if the absence of Rac2 impairs the ability of larvae to control fungal burden, we performed CFU counts on infected larvae at 1–4 dpi. However, as this experiment requires homogenizing and plating of the entire larva, we could not genotype larvae from a *rac2^+/−^* cross. Instead, we used a previously validated *rac2* morpholino oligonucleotide (MO) to inhibit Rac2 protein translation (25). To confirm the efficacy of *rac2* MO injection, we monitored neutrophil migration to a tail transection wound on a subset of larvae from each experiment, demonstrating that the *rac2* MO impaired neutrophil motility, a known phenotype of Rac2 knockdown (Fig. S1B) (25). In *rac2* MO-injected larvae, the fungal burden was higher than in control MO-injected larvae, although these differences were not statistically significant (Fig. 1B). It is also important to note that CFU counts may not accurately reflect *A. fumigatus* burden after spore germination and hyphal growth. In some cases, hyphae can be disrupted by homogenization and generate many CFUs while in other cases, hyphae can remain intact and form a single CFU. Thus, these data suggest that Rac2 might play a role in controlling fungal burden but are not conclusive.

### Rac2 deficiency does not impair the ability of macrophages to migrate to sites of *A. fumigatus* infection or internalize spores

**Fig 2:**
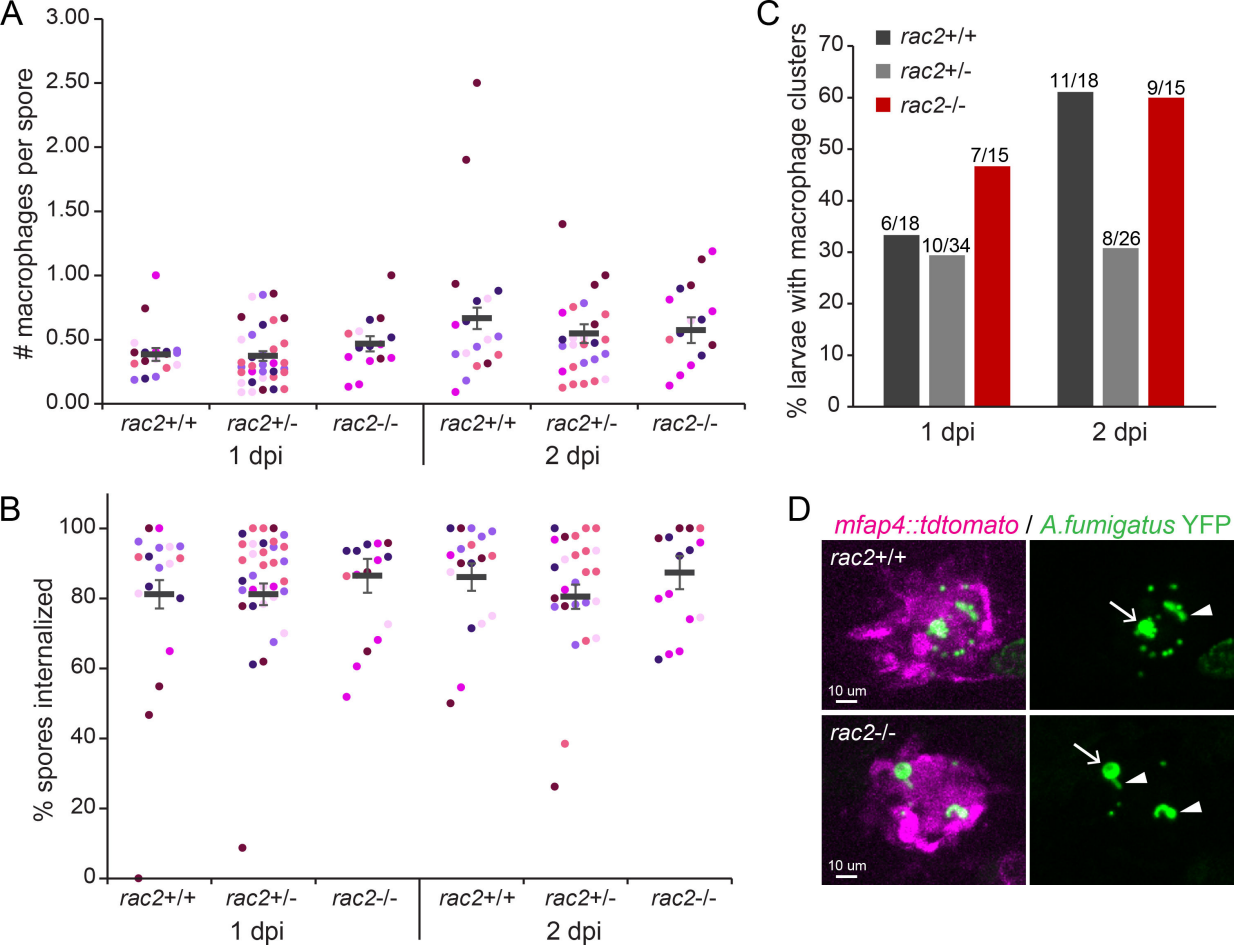
Rac2 is not required for macrophage migration to or phagocytosis of *A. fumigatus* spores. *rac2*^+/−^; *mfap4::tdtomato* adult fish were crossed to each other, and the resulting larvae were injected with YFP-expressing TBK1.1 (Af293) spores. Larvae were live imaged via confocal microscopy at 1 and 2 dpi and genotyped at the conclusion of the experiment. Average number of spores enumerated per larva via microscopy at 1 dpi = 60. Data represent 6 pooled replicates of 10–13 larvae per replicate. (A and B) The number of macrophages (*mfap4::tdtomato*) at the infection site and the number of spores present were counted, and the average number of macrophages per spore (**A**) and the percentage of spores found inside of macrophages (**B**) were calculated. Each symbol represents one larva, color coded by experiment. Lines represent emmeans ± SEM. *P* values calculated by ANOVA. (C) The presence of macrophage clusters was evaluated, and the percentage of larvae across all replicates with clusters is plotted. *P* values calculated by Fisher’s exact test. (D) Example images showing macrophage clustering around fungi. Both spore swelling (arrow) and germination (arrowhead) can be found from inside of macrophage clusters.

Rac2 expression and function are restricted to immune cells, and at this stage of larval zebrafish development, the primary immune cells present are neutrophils and macrophages (33). We previously demonstrated that the absence of Rac2 completely impairs the ability of neutrophils to migrate to sites of infection or wounding (24, 25), and thus, a major cause of the susceptibility of these larvae to *A. fumigatus* infection is likely to be a lack of neutrophil-mediated killing. However, the first cells to arrive to *A. fumigatus* infection are macrophages and these cells play a major role in phagocytosing *A. fumigatus* spores and inhibiting spore germination (13–15). Additionally, while the role of Rac2 in neutrophil function is more prominent, we previously reported that Rac2 deficiency can impair the function of macrophages in immune defense (24). Therefore, we performed repeated live confocal imaging at both 1 and 2 dpi on larvae resulting from a *rac2*^+/−^ cross that also carry the *mfap4::tdtomato* transgene labeling macrophages to determine if Rac2 is required in macrophages for these initial responses to *A. fumigatus* spore injection (Fig. 2). After infection with a YFP-expressing Af293-derived strain, the normalized number of macrophages recruited to and interacting with injected spores was not significantly different between *rac2^−/−^* and *rac2^+/−^* or *rac2^+/+^* larvae at either 1 dpi or 2 dpi, demonstrating that Rac2 is not required for macrophage migration to the site of *A. fumigatus* infection (Fig. 2A). After recruitment to the infection, macrophages phagocytose the majority of spores present (31), a process that can be dependent on Rac GTPases (22). We quantified the percentage of spores internalized by macrophages and found that ~80%–90% of spores were found inside of macrophages, with no significant differences between larvae of different *rac2* genotypes (Fig. 2B), demonstrating that in this infection scenario, Rac2 is not required for spore uptake by macrophages. These recruited macrophages can also form densely packed clusters, and we also did not observe a decrease in the formation of these clusters in *rac2^−/−^* larvae (Fig. 2C and D) (13).

### Rac2 is not required for spore trafficking to lysosomes or spore killing

**Fig 3:**
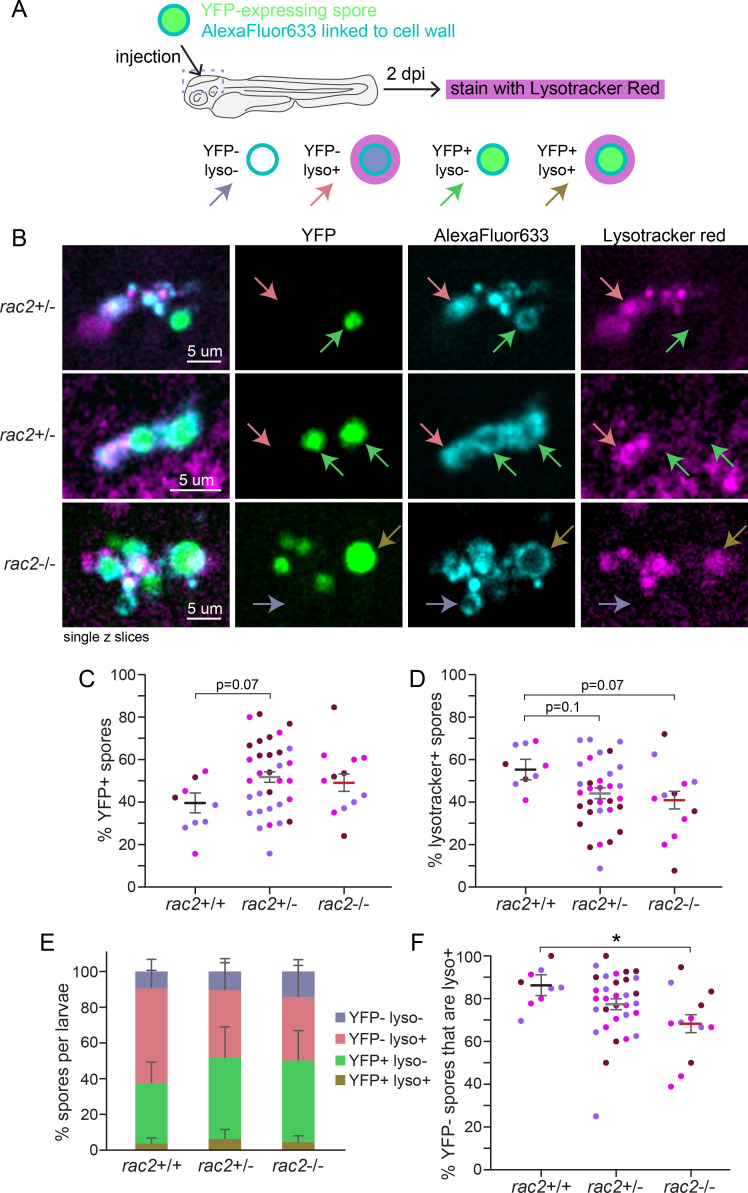
Rac2 is not required for spore acidification or killing. *rac2*^+/−^ adult fish were crossed to each other, and the resulting larvae were injected with YFP-expressing and AlexaFluor633-cell wall-conjugated TBK1.1 (Af293) spores. At 2 dpi, larvae were stained with Lysotracker Red and live imaged and then genotyped. Average number of spores enumerated per larva via microscopy at 2 dpi = 40. Data represent 3 pooled replicates of 17–18 larvae per replicate. (A) Experimental schematic. (B) Example images showing killed spores in non-acidified compartments (YFP−, AF+, lyso−; purple arrow), killed spores in acidified compartments (YFP−, AF+, lyso+; pink arrow), alive spores in non-acidified compartments (YFP+, AF+, lyso−; green arrow), and alive spores in acidified compartments (YFP+, AF+, lyso+; brown arrow). Each image shows a single z slice from a confocal image stack. (C) The percentage of alive (YFP+) spores was calculated for each larva. (D) The percentage of spores present in acidified compartments (lyso+) was calculated for each larva. (E) The average percentage of spores in each type of compartment was calculated across all larvae. (F) The percentage of killed (YFP−) spores that are found in acidified compartments (lyso+) was calculated for each larva. For (C, D, and F) each symbol represents one larva, color coded by replicate. For C–F, lines represent emmeans ± SEM. *P* values were calculated by ANOVA.

After phagocytosis, macrophages traffic spores to phagolysosomes for killing (15). As a GTPase involved in actin polymerization, we hypothesized that Rac2 deficiency alters the trafficking of spores to acidified compartments and therefore the killing of spores. To test this, we again crossed *rac2*^+/−^ zebrafish to each other and infected larvae with a YFP-expressing Af293-derived strain coated in AlexaFluor633 cell wall tracer, an established method for evaluating spore killing (13, 34). At 2 dpi, we stained larvae with Lysotracker Red and performed confocal imaging (Fig. 3A). Consistent with previous results, we found both alive (YFP+, AF+) and dead (YFP-, AF+) spores in control larvae (Fig. 3B) (13). While there was a slight trend towards a higher percentage of alive (YFP+) spores per larvae in *rac2^−/−^* versus *rac2^+/+^* larvae, this difference was not statistically significant, suggesting that Rac2 is not required for spore killing (Fig. 3C). We also quantified the percentage of spores in acidified compartments, as marked by Lysotracker Red staining. Again, while there was a trend toward fewer acidified spores in *rac2^−/−^* larvae compared with *rac2^+/+^* larvae, this difference was not statistically significant (Fig. 3D). While most killed spores were found in acidified compartments (Fig. 3B, pink arrow) and most alive spores were not acidified (Fig. 3B, green arrow), we also identified some spores that were dead but not in acidified compartments (Fig. 3B, purple arrow) and that were in acidified compartments but still alive (Fig. 3B, brown arrow). The distribution of spores across these different categories was similar between all three genotypes (Fig. 3E; Fig. S2). However, Rac2 may promote killing of spores through trafficking to acidified compartments. Focusing specifically on killed spores (YFP−, AF+), a statistically significantly lower percentage of these spores was in acidified compartments in *rac2^−/−^* larvae compared with *rac2^+/+^* larvae (Fig. 3F). Altogether, data from this experiment suggest that macrophages can utilize multiple mechanisms to kill spores, either dependent or independent of acidification. While Rac2 is not required for overall macrophage killing ability, it may promote acidification-mediated killing.

### Rac2 functions in macrophages to inhibit spore germination

As only ~50% of spores are killed by 2 dpi, some of these spores eventually germinate, and we observed both spore swelling and germination from the inside of both single macrophages and macrophage clusters, in larvae of all *rac2* genotypes (Fig. 2D). Macrophages play an important role in inhibiting fungal spore germination, but quantification of this effect is complicated by the ability of neutrophils to kill fungi post-germination. To eliminate the function of neutrophils in all larvae and focus specifically on the function of Rac2 in macrophages in controlling germination and invasive hyphae, we crossed the *rac2* mutant line to the neutrophil-defective *mpx::mCherry-2A-rac2^D57N^* transgenic line (hereafter referred to as *mpx::rac2^D57N^*) which expresses a dominant-negative copy of Rac2 under a neutrophil-specific promoter. We then crossed *rac2^+/−^*; *mpx::rac2^D57N^* adults to each other and infected the resulting larvae with a YFP-expressing Af293-derived strain of *A. fumigatus* and monitored fungal germination and hyphal growth in larvae for 5 days by repeated, daily live confocal imaging (Fig. 3). In *rac2^+/+^* and *rac2^+/−^* larvae, we observed germination in ~60% and invasive hyphae in ~30% of larvae at 3 dpi (Fig. 3A and B). However, in *rac2^−/−^* larvae, >90% of larvae experienced fungal spore germination and >70% experienced invasive hyphal growth by 3 dpi (Fig. 3A and B). Comparison of the rate of appearance of both germination and invasive hyphae demonstrates that *rac2^−/−^* larvae are 2.6–3.1 times more likely to experience fungal growth relative to their wild-type and heterozygote siblings, a statistically significant difference. These data demonstrate that Rac2 in macrophages promotes the control of *A. fumigatus* spore germination.

### Rac2 functions in macrophages to inhibit extracellular hyphal growth

**Fig 4:**
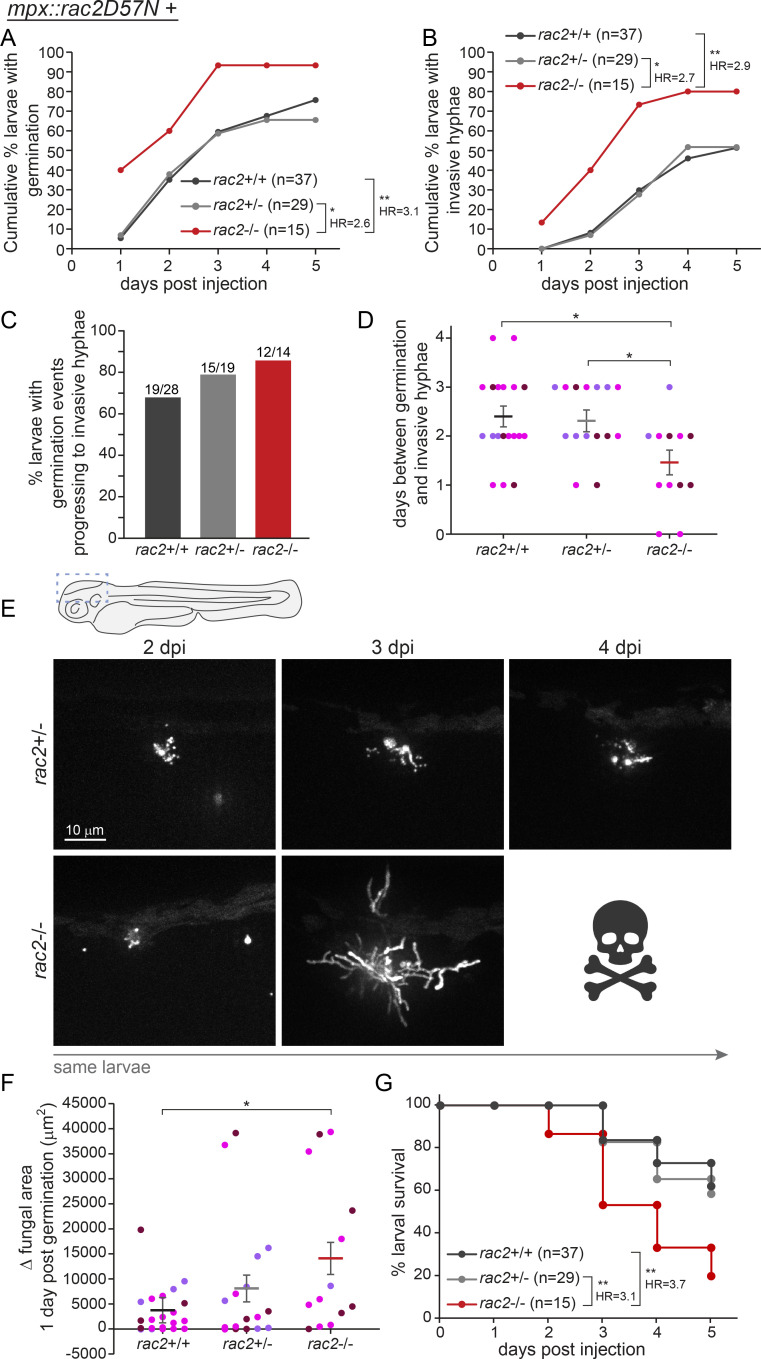
Macrophages control spore germination and extracellular hyphal growth through Rac2 function. *rac2*^+/–^; *mpx::mCherry-2A-rac2^D57N^* adult fish were crossed to each other, and the resulting larvae were injected with YFP-expressing TBK1.1 (Af293) spores. Larvae were live imaged via confocal microscopy at 1–5 dpi and genotyped at the conclusion of the experiment. All larvae in the experiment carry the *mpx::rac2^D57N^* transgene. Data represent 3 pooled replicates of 22–31 larvae per replicate. (A and B) The cumulative percentage of larvae experiencing germination (**A**) and invasive hyphae (**B**) was calculated. *P* values and hazard ratios were calculated by Cox proportional hazard analysis. (C) Of larvae experiencing germination, the percentage that progress to invasive hyphae was calculated. *P* values calculated by Fisher’s exact test. (D) Of larvae that experience germination and invasive hyphae, the number of days between the observance of these forms of growth was calculated. Each symbol represents one larva, color coded by experiment; lines represent emmeans ± SEM. *P* values calculated by ANOVA. (E) Example images showing germination in larvae at 2 dpi and subsequent control of fungal growth (top) or invasive hyphae development and death (bottom). (F) In larvae that experience germination, the fungal (YFP+) area was measured at both 0 and 1 dpg. Each symbol represents one larva, color coded by experiment; lines represent emmeans ± SEM. *P* values calculated by ANOVA. (G) Survival of larvae was monitored for the 5 days of imaging. *P* values and hazard ratios calculated by Cox proportional hazard analysis.

We were also interested in whether Rac2 plays a role in the ability of macrophages to control extracellular hyphal growth after germination has occurred. While neutrophils are regarded as the primary innate immune cell type capable of killing large extracellular pathogens, we have previously observed macrophages also coating hyphae in larval zebrafish, suggesting that these cells may have some ability to inhibit hyphal growth (30). We therefore quantified fungal growth further in this experiment. As discussed above, we observed that a higher rate of *rac2^−/−^* larvae experienced invasive hyphal growth than *rac2^+/+^* or *rac^+/−^* larvae (Fig. 4B). However, this difference could simply be due to a higher rate of initial germination. To specifically investigate post-germination control of fungal growth, we focused only on larvae in which germination had already occurred. Of these larvae, a higher percentage of *rac2^−/−^* larvae progressed to invasive hyphae compared with their sibling controls (Fig. 4C). Additionally, for those that did progress to invasive hyphae, this occurred significantly faster in *rac2^−/−^* larvae, after an average of ~1.5 days, compared with *rac2^+/+^* or *rac2^+/−^* larvae in which this progression took an average of ~2.3 days (Fig. 4D). In particular, several control larvae experienced germination relatively early after spore injection but were able to control fungal growth for multiple days afterwards (Fig. 4E). On the other hand, many *rac2^−/−^* larvae had the same type of initial germination which quickly progressed to invasive hyphae, causing mortality (Fig. 4E). From our confocal images, we quantified the amount of fungal growth by measuring the YFP+ area on the day of germination compared with 1 day later, finding that in *rac2^−/−^* larvae, there was significantly more fungal growth compared with wild-type larvae 1 day post germination (dpg) (Fig. 4F). All of the larvae in this experiment had defective neutrophils that were unable to migrate to the site of infection, and thus, these data demonstrate that macrophages alone are able to partially control *A. fumigatus* extracellular hyphal growth and that Rac2 functions in these macrophages to promote hyphal control. We also tracked the survival of these imaged larvae, confirming that even in the absence of functional neutrophils, Rac2 deficiency leads to significantly decreased larval survival after infection (Fig. 4G).

### Re-expression of Rac2 specifically in macrophages promotes host survival and control of fungal growth

**Fig 5:**
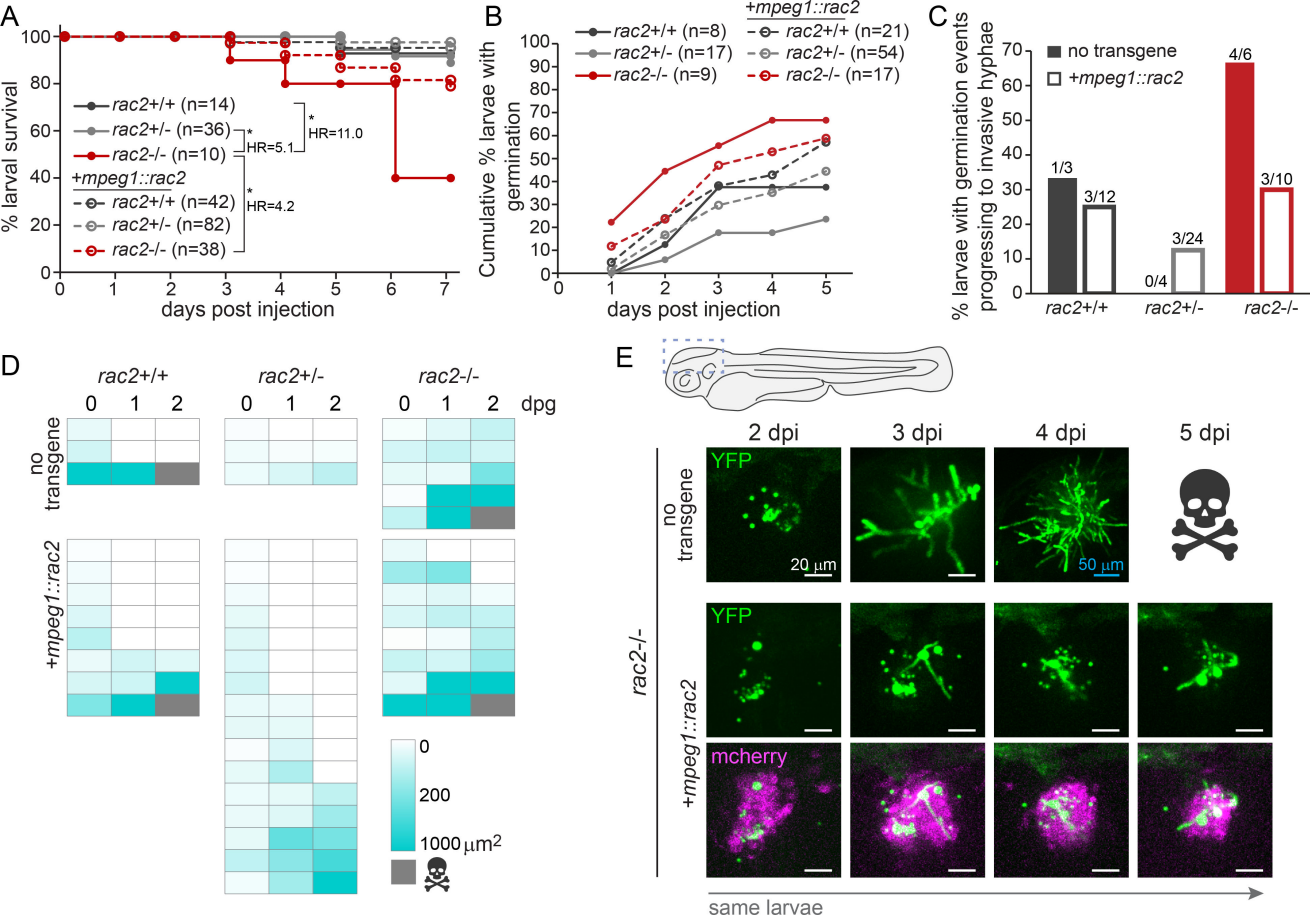
Macrophage-expressed Rac2 is sufficient to promote host survival against invasive *A. fumigatus* growth. *rac2*^+/−^ and *rac2*^+/−^; *mpeg1::mCherry-2A-rac2* adults were crossed to each other, and the resulting larvae were injected with YFP-expressing TBK1.1 (Af293) spores. (A) Survival was monitored for 7 dpi, and larvae were genotyped at the conclusion of the experiment for both the endogenous *rac2* genotype and presence of the transgene. Average injection CFUs = 45. Data represent three pooled replicates. *P* values and hazard ratios were calculated by Cox proportional hazard analysis. (B–E) Larvae were live imaged via confocal microscopy at 1–5 dpi and genotyped at the conclusion of the experiment. Data represent 3 pooled replicates of 42 larvae per replicate. (B) The cumulative percentage of larvae experiencing germination was calculated. *P* values and hazard ratios calculated by Cox proportional hazard analysis. (C) Of larvae experiencing germination, the percentage that progress to invasive hyphae was calculated. *P* values calculated by Fisher’s exact test. (D) Fungal (YFP+) areas 0, 1, and 2 dpg were measured in larvae that experience germination at 3 dpi and displayed in a heatmap. (E) Example images showing germination in larvae at 3 dpi and subsequent invasive hyphae development and death (top) or control of fungal growth in larvae re-expressing Rac2 and mCherry in macrophages (bottom).

We also wondered if macrophage-specific rescue of Rac2 expression in *rac2^−/−^* larvae could promote larval survival and control of fungal growth after infection. To that end, we crossed the *rac2* mutant line to a *mpeg1::mCherry-2A-rac2* transgenic line (hereafter referred to as *mpeg1::rac2*) which expresses a wild-type copy of Rac2 under a macrophage-specific promoter (24). We then crossed *rac2^+/−^*; *mpeg1::rac2* adults with *rac2^+/−^* larvae and infected the resulting larvae with an Af293-derived strain of *A. fumigatus*, assayed for survival, and genotyped larvae at the conclusion of the experiment for both endogenous *rac2* genotype and the presence of the transgene. As observed previously, *rac2^−/−^* larvae succumbed to the infection at a significantly higher rate than *rac2^+/+^* or *rac2^+/−^* larvae (Fig. 5A). However, rescue of Rac2 expression only in macrophages improved larval survival to levels almost indistinguishable from control larvae, a statistically significant difference (Fig. 5A). To monitor fungal germination and hyphal growth, we performed the same cross, infected larvae with a YFP-expressing Af293-derived strain of *A. fumigatus* and performed repeated, daily live confocal imaging for 5 days. As observed previously, *rac2^−/−^* larvae had higher rates of spore germination than control siblings (Fig. 5B). This germination was decreased in *rac2^−/−^* larvae re-expressing Rac2 in macrophages (Fig. 5B). Additionally, while 4 of 6 *rac2^−/−^* larvae that experienced germination progressed to invasive hyphae, this only occurred in 3 of the 10 *rac2^−/−^* larvae re-expressing Rac2 in macrophages (Fig. 5C). While these differences in germination and invasive hyphal development were not statistically significant, these data suggest that re-expression of Rac2 in macrophages promotes infected larval survival through increased control of fungal burden. Quantification of the fungal area in all larvae that experienced germination by 3 dpi demonstrates that more larvae re-expressing Rac2 in macrophages were able to control initial fungal growth and prevent invasive growth (Fig. 5D and E). These data are consistent with the conclusion that Rac2 plays a significant role in macrophages to promote control of extracellular fungal growth of *A. fumigatus* and to promote host survival.

## DISCUSSION

Neutrophils are generally regarded as the most important innate immune cell type for control of *A. fumigatus* and other invasive fungal infections (35, 36) and are a common target of host-directed therapies aiming to boost innate immune responses to infection in immunocompromised patients (37). Macrophages, which are present basally in tissues, are another potential target, but whether macrophages can function against invasive forms of fungal infections like *A. fumigatus* has not been clear. Here, using a larval zebrafish host, we demonstrate that in the absence of functional neutrophils, macrophages are able to control the growth of hyphae. While this control is less effective than neutrophil-mediated killing of hyphae, macrophages do significantly slow down hyphal growth and significantly prolong host survival. Our study was performed with a strain of *A. fumigatus* derived from Af293, a human patient isolate that has a relatively slow germination and growth rate (13). In infections with faster germinating strains, it is possible either that macrophages are less able to control fungal growth or that macrophages are even more important to keep this growth in check.

What pathways and mechanisms macrophages use to control hyphal growth is still not known but require functional Rac2. One possibility is that macrophages release ROS extracellularly through the phagocyte oxidase (PHOX) complex. While the PHOX complex is often thought of as localizing to phagosomes, it can also localize to the plasma membrane (38), and extracellular release of ROS by neutrophils can kill hyphae (10). The PHOX complex requires Rac GTPase activity to produce ROS, and Rac2 can function in this complex (22, 39, 40). Neutrophils can produce extracellular traps in response to hyphae (9), and in a few cases, macrophages have been found to form similar structures (41). However, if macrophages can generate these structures against *A. fumigatus* and if Rac2 is required for their formation are unknown. Through timelapse imaging, we have also observed macrophages coating and wrapping around hyphae (30), similar to the “frustrated phagocytosis” observed when macrophages encounter growing hyphae of *Candida albicans* (42). This frustrated phagocytosis involves formation of an actin cuff, and Rac2 may promote this process through its activation of actin polymerization. Additionally, Rac2-deficient murine bone marrow-derived macrophages have lowered COX2 expression (19). We recently reported that a COX-PGE2 signaling axis promotes control of germination and extracellular growth of fungus by both macrophages and neutrophils (43), and this may be another mechanism through which Rac2 promotes control of hyphal growth by macrophages.

Surprisingly, we find that Rac2 is not required in macrophages for earlier responses to the infection, including cell migration, phagocytosis, phagosomal acidification, and spore killing. Phagocytosis *in vitro* does involve actin structures and can be blocked by cytochalasin D treatment (15, 16), but it could be that a different Rac GTPase, like Rac1 or Cdc42, can perform this function in the absence of Rac2 (17). We did find that significantly fewer killed spores were in acidified compartments in *rac2* mutant larvae compared with wild-type larvae, suggesting that Rac2 might promote phagosomal trafficking to acidified compartments but that there are multiple other mechanisms that these cells can use to kill spores, including ROS production and all of the antimicrobial peptides and degradative enzymes present in lysosomes. However, we again find that the spore killing of macrophages in the larval zebrafish model is very low, with only ~50% of spores killed by 2 dpi (30, 31). This is in contrast to some reports of spore killing by macrophages *in vitro* (15, 44), and it is unclear why these *in vivo* macrophages fail to kill spores efficiently. We find that spore killing is correlated with spore acidification, and we hypothesize that non-killed spores aren’t trafficked correctly by these cells. It is known that pksP-generated melanin pigment can inhibit phagolysosome fusion in macrophages, and that likely happens in zebrafish as well (45). It was also recently reported that *A. fumigatus* spores utilize the human p11 protein to escape killing in epithelial cells, but it is unclear if this also occurs in professional phagocytes like macrophages (46). Additionally, we also observe some spores that are killed without being acidified and the different killing mechanisms that are used against spores *in vivo* by macrophages are not fully known. Rac2 is required in macrophages to inhibit spore germination into hyphae, which also indicates that acidification is not the primary mechanism through which macrophages inhibit this process either.

Altogether, we report that Rac2 functions in macrophages against *A. fumigatus* and that the main function of Rac2 is to promote control of spore germination and control of subsequent extracellular hyphal growth. Future studies are required to further understand the microbicidal functions of macrophages against both intracellular *A. fumigatus* spores and extracellular hyphae and how Rac2 promotes these functions.

## MATERIALS AND METHODS

### Zebrafish lines and maintenance

Adult and larval zebrafish were maintained and handled according to protocols approved by the Clemson University Institutional Animal Care and Use Committee (AUP2021-0109, AUP2022-0093, and AUP2022-0111). Adult zebrafish were maintained at 28°C in a 14-/10-hour light/dark cycle. All mutant and transgenic fish lines used in this study are listed in Table 1 and were maintained in the AB background. Adults were spawned naturally, and embryos were kept in E3 medium with methylene blue at 28°C. Embryos were manually dechorionated and anesthetized in 0.3 mg/mL buffered tricaine prior to any experimental manipulations. The *rac2^uwm32^* line was maintained as heterozygotes by out-crossing to wild-type or transgenic lines. *rac2^+/−^* larvae were crossed to each other for experiments to generate *rac2^+/+^*, *rac2^+/−^*, and *rac2^−/−^* progeny. Experiments were then performed blind, genomic DNA was isolated from larvae, and larvae were genotyped at the conclusion of the experiment. Genotyping was performed as previously described (24) except that for amplification of the target site, a different reverse primer was used that binds to an intron at the endogenous *rac2* locus and therefore does not amplify any *rac2* transgenes present in the genome (F: 5′ CACTGCCCTTCCACTCCAAT 3′ , R: 5′ GACAAATCCAGCTCTGCCAC 3′). PCR was also done to confirm the presence of the *mpeg1::mCherry-2A-rac2* transgene in larvae with primers that bind to the sequences of the *mpeg1* promoter (F: 5′ TGCGTCATCAAAACCATTCTGA 3′) and *mCherry* (R: 5′ ACCTTGAAGCGCATGAACTC 3′).

**TABLE 1:** Zebrafish lines used in this study

Line name	Purpose	Reference
*rac2^uwm32^*	Global loss of function mutation in *rac2* gene	(24)
*Tg(mfap4::tdtomato*)	Transgenic expression of tdtomato in macrophages	(47)
*Tg(mpx::mCherry-2A-rac2^D57N^*)	Transgenic expression of dominant-negative Rac2 in neutrophils	(25)
*Tg(mpeg1::mCherry-2A-rac2*)	Transgenic expression of wild-type Rac2 in macrophages	(24)

### *rac2* morpholino injections

A *rac2* MO (ZFIN MO1-rac2; 5′-CCACCACACACTTTATTGCTTGCAT-3′) (GeneTools) was previously published and validated (25). A 1 mM stock solution in water was kept at 4°C. For injections, the stock was diluted to 0.1 mM in water with 0.1% filter-sterilized phenol red and 0.5× CutSmart Buffer (New England Biolabs). A standard control MO (GeneTools) was used as an injection control at the same concentration. A microinjection setup (BTX, Microject 1000A) supplied with pressure injector, micromanipulator (Narishige), micropipet holder, footswitch, and compressed nitrogen gas was used to inject 3 nl of injection mix into the yolk of 1–2 cell stage embryos. The efficiency of *rac2* knockdown was determined by performing tail transection and sudan black staining to quantify neutrophil motility, as previously described (24), on injected larvae from the same clutch for each experimental replicate.

### *Aspergillus fumigatus* strains and spore preparation for injections

All experiments were done with an Af293-derived strain expressing YFP (TBK1.1), which behaves like the parental strain in larval zebrafish (13, 31). Spores were isolated for injection as previously described (31, 43). On 10-cm solid glucose minimal media (GMM) plates, 10^6^ spores were spread and grown at 37°C for 3–4 days. Spores were harvested by scraping with a disposable L-spreader into 50 mL sterile water with 0.01% Tween and passed through two layers of sterile miracloth into a 50-mL conical tube and pelleted by centrifugation at 900 *g* for 10 min. The pellet was washed in 50 mL of sterile PBS. After centrifugation again, the pellet was resuspended in 5 mL PBS, filtered through sterile miracloth again, the spore concentration was determined with a hemacytometer, and a final stock of 1.5 × 10^8^ spores/mL was made in PBS and stored at 4°C for up to ~1 month. For live-dead staining, spores were coated with AlexaFluor633 as described previously (13, 34). Briefly, spores were incubated with biotin-XX, SSE (Molecular Probes) in the presence of 0.05 M NaHCO_3_ at 4°C for 1.5–2 hours. Spores were then washed with 100 mM Tris-HCl pH 8.0 to deactivate free-floating biotin and then incubated with streptavidin-AlexaFluor633 (Invitrogen) in PBS, washed, and stored at 4°C for up to ~2 weeks.

### Larval zebrafish hindbrain microinjection

Larvae were injected with spores as described previously (32). Spore suspensions (1.5 × 10^8^ spores/mL) were mixed with filter-sterilized 1% phenol red to achieve a final spore concentration of 1 × 10^8^/mL. Anesthetized 2 dpf larvae were placed on an agarose plate on their lateral side for injection. A microinjection setup (Applied Scientific Instrumentation, MPPI-3) supplied with a pressure injector, micromanipulator (Narishige), micropipet holder, footswitch, back pressure unit, and compressed filtered air was used to inject ~50 spores into the hindbrain ventricle of each larva. After injection, larvae were rinsed at least twice with E3 without methylene blue to remove tricaine and any free spores. For all experiments, pooled data from at least three independent replicates were generated and Ns are presented in each figure or figure legend.

### Survival analysis

For survival analyses, larvae were placed in individual wells of a 96-well tissue culture-treated plate and monitored for 7 dpi. From the pool of injected larvae, eight were randomly selected and homogenized for CFU plating as described below to monitor actual injection dose which is reported in figure legends. Survival data were analyzed in R version 3.5.2 by Cox proportional hazard regression as previously described (13, 43). In these analyses, experimental replicate is included as a group variable and hazard ratios are calculated. The hazard ratio can be interpreted as the relative death rate and represents the relative instantaneous risk of death throughout the experiment between two conditions.

### CFU analysis

To monitor fungal burden, larvae were placed singly in 1.7 mL microcentrifuge tubes in 90 µL PBS containing 1 mg/mL ampicillin and 0.5 mg/mL kanamycin, homogenized in a TissueLyser (Qiagen) at 1,800 oscillations/minute (30 Hz) for 6 minutes. Samples were centrifuged at 17,000 *g* for 30 seconds, spread undiluted on GMM plates, and incubated at 37°C for 3 days. For each day, condition, and replicate, eight larvae were plated. In a few cases, only 4–7 larvae remained at 4 dpi and only this number of larvae was plated. The number of fungal colonies were counted and normalized to the average CFU burden at 0 dpi to calculate the percent initial spore burden. Data were analyzed in R version 3.5.2 by ANOVA. For each condition, estimated marginal means (emmeans) and SEM were calculated and pairwise comparisons were performed with Tukey’s adjustment.

### Live imaging analysis

For all imaging experiments, larvae were treated with 200 µM N-phenylthiourea (PTU) from 24 hours post fertilization onwards. For daily imaging experiments, larvae were placed in individual wells of a 48-well tissue culture-treated plate after injection. On each day, larvae were removed one at a time, anesthetized in tricaine, and transferred to a zWEDGI device (48). After imaging, larvae were rinsed in E3 with PTU and returned to the same well. For live-dead staining, larvae were placed in 6-cm milk-treated petri dishes after injection. At 2 dpi, larvae were stained with 10 µM Lysotracker Red DND-99 (Invitrogen) at 28°C for 1 hour prior to imaging. Larvae were rinsed twice in E3 prior to anesthesia with tricaine and imaging in the same zWEDGI device. Larvae were imaged using a Zeiss Cell Observer Spinning Disk confocal microscope on a Axio Observer 7 microscope stand with a confocal scanhead (Yokogawa CSU-X) and a Photometrics Evolve 512 EMCCD camera. A Plan-Apochromat 10× objective (0.3 NA), Plan-Apochromat 20× objective (0.8 NA), or EC Plan-Neofluar 40× objective (0.75 NA) and ZEN software were used to acquire Z-stack images of the hindbrain area.

All images were analyzed with ImageJ/Fiji (49). Macrophage number, spore internalization, live versus dead spores, and lysotracker staining were quantified manually with the Cell Counter plugin. The presence of macrophage cluster, germination, and invasive hyphae were also manually counted. Germination was defined as a hyphal fragment longer than the diameter of the spore, and invasive hyphae was defined as hyphae containing at least one branching event. The fungal area after germination was measured by fluorescent intensity thresholding from maximum intensity Z projections encompassing the entire fungal growth. All displayed images are processed with bilinear interpolation to increase the pixel density twofold or fourfold. Cumulative appearance of germination or invasive hyphae was analyzed by Cox proportional hazard regression in R version 3.5.2. Macrophage number, spore internalization, spore killing, lysotracker staining, days between germination and invasive hyphae, and fungal area were analyzed with ANOVA in R version 3.5.2. For each condition, estimated marginal means (emmeans) and SEM were calculated and pairwise comparisons were performed with Tukey’s adjustment. The percentage of larvae with macrophage clusters or with germination progressing to invasive hyphae was analyzed with Fisher’s exact test in R version 3.5.2.

## SUPPLEMENTAL MATERIAL

Supplemental figures iai.00380-23-s0001.pdf Figures S1 and S2.

ASM does not own the copyrights to Supplemental Material that may be linked to, or accessed through, an article. The authors have granted ASM a non-exclusive, world-wide license to publish the Supplemental Material files. Please contact the corresponding author directly for reuse.
