# Supplementary material for: Macrophages inhibit extracellular hyphal growth of A. fumigatus through Rac2 GTPase signaling
Source: Infect Immun. 2024 Jan 3;92(2):e00380-23. doi: 10.1128/iai.00380-23 (PMC10863406; doi:10.1128/iai.00380-23)
Supplement: Supplemental figures — Figures S1 and S2. [file iai.00380-23-s0001.pdf]

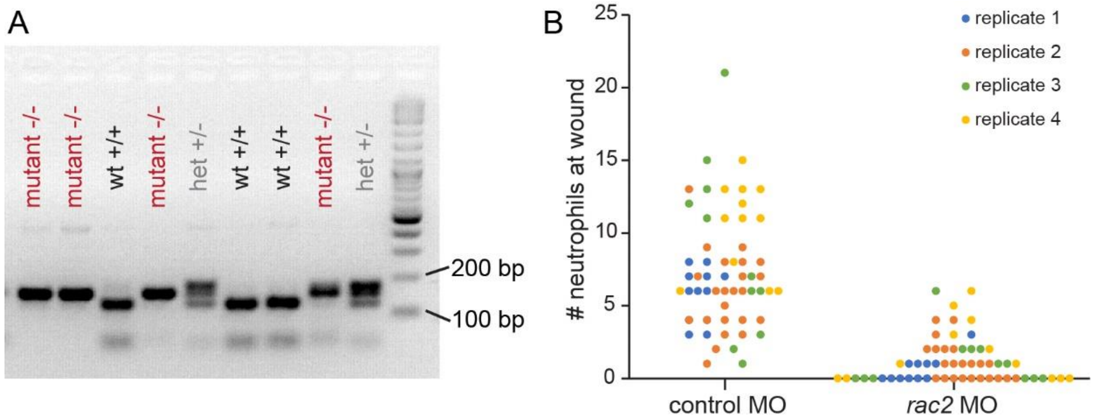

Supp Fig 1. Genotyping and phenotyping of *rac2* mutant and knockdown larvae. A. Example genotyping gel for *rac2* mutant line. PCR amplifies 164 bp around the mutation site. The mutation abolishes a HindIII restriction site that in wild-type copies of DNA results in band sizes of 39 and 125 bp after restriction digest. **B.** A subset of larvae from each experiment treated with a *rac2* or standard control morpholino (MO) were tail wounded by transection at 2 dpf. At ~2 hpw, larvae were fixed, neutrophils were stained with sudan black, and the number of neutrophils responding to the wound were counted via brightfield microscopy. Each symbol represents one larva, color-coded by experiment.

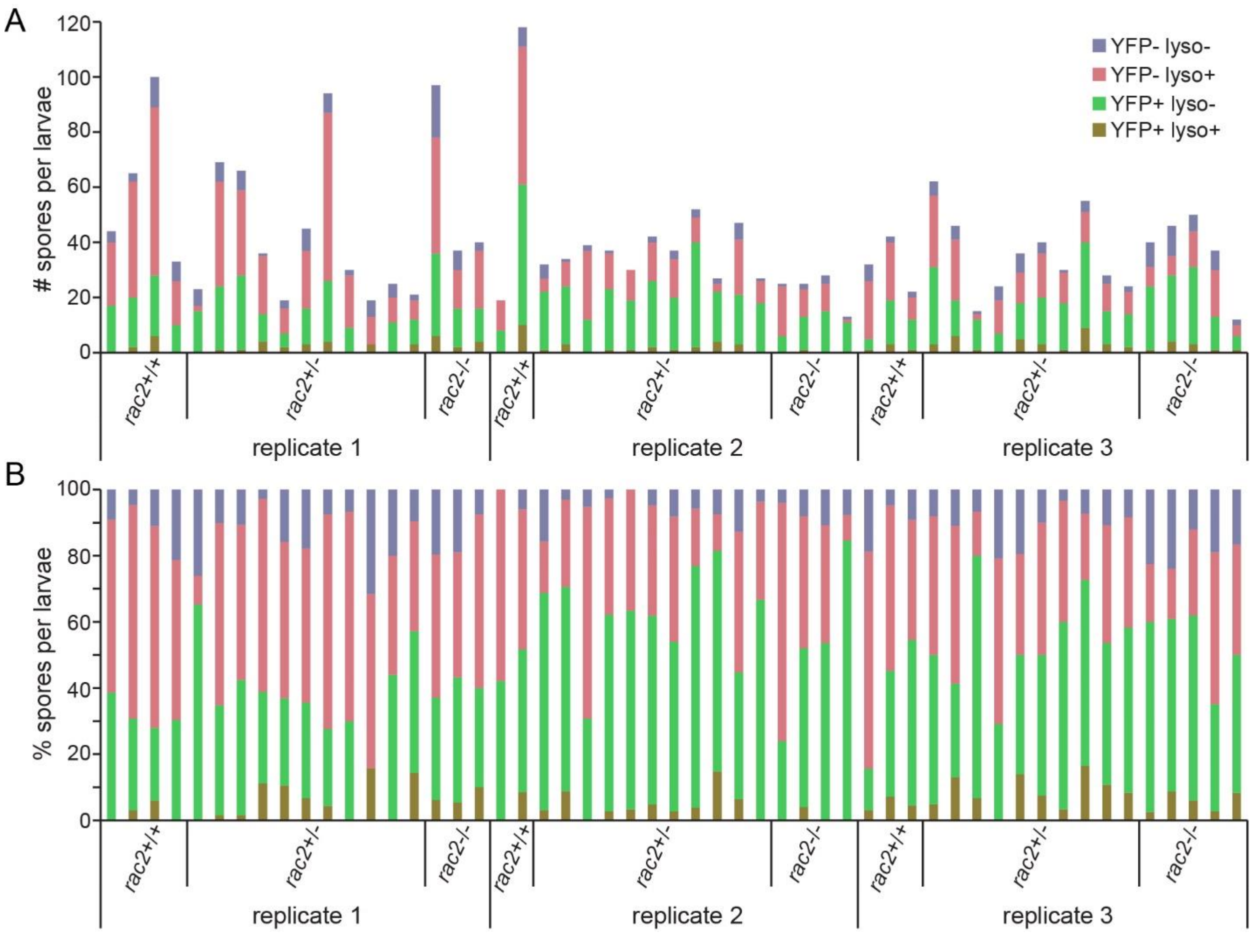

Supp Fig 2. Percentage of killed and acidified spores in individual larvae across all replicates. Larvae resulting from a *rac2*$^{+/-}$ in-cross were injected with YFP-expressing and AlexaFluor633-cell wall conjugated TBK1.1 (Af293) spores. At 2 dpi, larvae were stained with Lysotracker red and live imaged and then genotyped. Data shown averaged in Fig 3E is here displayed for each individual larva. **A.** The number of spores in each type of compartment was calculated for each larva. **B.** The percentage of spores in each type of compartment was calculated for each larva.
